# Fluorescence-guided imaging for resection margin evaluation in head and neck cancer patients using cetuximab-800CW: A quantitative dose-escalation study

**DOI:** 10.7150/thno.43227

**Published:** 2020-03-04

**Authors:** Floris Jan Voskuil, Steven Jakob de Jongh, Wouter Tjerk Rudolph Hooghiemstra, Matthijs David Linssen, Pieter Jan Steinkamp, Sebastiaan Antonius Hendrik Johannes de Visscher, Kees-Pieter Schepman, Sjoerd Geert Elias, Gert-Jan Meersma, Pascal Klaas Christiaan Jonker, Jan Johannes Doff, Annelies Jorritsma-Smit, Wouter Bastiaan Nagengast, Bert van der Vegt, Dominic James Robinson, Gooitzen Michell van Dam, Max Johannes Hendrikus Witjes

**Affiliations:** 1 Department of Oral & Maxillofacial Surgery, University of Groningen, University Medical Center Groningen, the Netherlands; 2 Department of Gastroenterology and Hepatology, University of Groningen, University Medical Center Groningen, the Netherlands; 3 Department of Clinical Pharmacy and Pharmacology, University Medical Center Groningen, The Netherlands; 4 Department of Surgery and Medical Imaging Center, University of Groningen, University Medical Center Groningen, the Netherlands; 5 Department of Epidemiology, Julius Center for Health Sciences and Primary Care, University Medical Center Utrecht, Utrecht University, The Netherlands.; 6 Department of Pathology & Medical Biology, University of Groningen, University Medical Center Groningen, the Netherlands; 7 Center for Optical Diagnostics and Therapy, Department of Otorhinolaryngology and Head and Neck Surgery, Erasmus MC Cancer Institute, Rotterdam, the Netherlands; 8 TRACER Europe B.V. / AxerlaRx, Groningen, The Netherlands

**Keywords:** fluorescence-guided imaging, intrinsic fluorescence, MDSFR/SFF spectroscopy, cetuximab-800CW, head and neck cancer

## Abstract

Tumor-positive resection margins are present in up to 23% of head and neck cancer (HNC) surgeries, as intraoperative techniques for *real-time* evaluation of the resection margins are lacking. In this study, we investigated the safety and potential clinical value of fluorescence-guided imaging (FGI) for resection margin evaluation in HNC patients. We determined the optimal cetuximab-800CW dose by quantification of intrinsic fluorescence values using multi-diameter single-fiber reflectance, single-fiber fluorescence (MDSFR/SFF) spectroscopy.

**Methods**: Five cohorts of three HNC patients received cetuximab-800CW systemically: three single dose cohorts (10, 25, 50 mg) and two cohorts pre-dosed with 75 mg unlabeled cetuximab (15 or 25 mg). Fluorescence visualization and MDSFR/SFF spectroscopy quantification was performed and were correlated to histopathology.

**Results**: There were no study-related adverse events higher than Common Terminology Criteria for Adverse Events grade-II. Quantification of intrinsic fluorescence values showed a dose-dependent increase in background fluorescence in the single dose cohorts (*p<0.001, p<0.001*), which remained consistently low in the pre-dosed cohorts *(p=0.6808)*. Resection margin status was evaluated with a sensitivity of 100% (4/4 tumor-positive margins) and specificity of 91% (10/11 tumor-negative margins).

**Conclusion**: A pre-dose of 75 mg unlabeled cetuximab followed by 15 mg cetuximab-800CW was considered the optimal dose based on safety, fluorescence visualization and quantification of intrinsic fluorescence values. We were able to use a lower dose cetuximab-800CW than previously described, while remaining a high sensitivity for tumor detection due to application of equipment optimized for IRDye800CW detection, which was validated by quantification of intrinsic fluorescence values.

## Introduction

Head and neck cancer (HNC) is the sixth most common cancer worldwide, with an incidence of over 550,000 and around 300,000 cancer-related deaths each year 1. The most common histological subtype is head and neck squamous cell cancer (HNSCC), accounting for more than 90% of all HNCs. Depending on anatomical location, the primary treatment of HNSCC consists of surgical resection or (chemo)radiotherapy 2-7. In case of surgical treatment, tumor-negative resection margins are essential for local control and consequently improve survival. A tumor-positive resection margin in head and neck oncology is generally defined as viable tumor cells within 1 mm of the resection margin. Tumor-negative resection margins reduce the need for adjuvant treatment, while a tumor-positive resection margin often involves treatment with cisplatin-based chemo-radiation, which severely increases the risk of sequelae.

Despite improving pre- and intraoperative imaging techniques, no accurate clinically approved tools are available to discriminate between tumor and surrounding non-tumor tissue for *real-time* margin assessment during surgery. Consequently, surgeons rely on their experience and visual and tactile information alone. Due to the intrinsic limitations of the provided information, this contributes to a tumor-positive resection margin rate that is reported to be as high as 23% in HNSCC 8-10.

With the development of clinical grade small peptides and antibodies conjugated to fluorescent dyes, the field of fluorescence-guided imaging (FGI) has rapidly evolved and expanded to application in several solid tumor types 11-14. FGI has the potential to provide *real-time* feedback on increased receptor expression in cancerous tissue and highlighting these cancer cells by binding of fluorescent labelled tracers, making it an interesting technique for intraoperative margin evaluation. Since the first clinical study on FGI in 2011, in which a conjugate of folate-FITC was used to visualize peritoneal metastasis of ovarian cancer, several studies in different tumor types and organs have been performed that further demonstrate the potential of FGI for discrimination between tumor and healthy tissue 13,15-19.

A promising target for targeted-FGI in HNSCC patients is the endothelial growth factor receptor (EGFR). Overexpression of EGFR in HNSCC is present in up to 90% of the cases and is associated with higher local recurrence and poorer survival rates 20,21. Two early phase clinical trials on FGI in HNSCC patients have already been performed, investigating a monoclonal antibody (cetuximab and panitumumab respectively) targeting EGFR, which was conjugated to the near-infrared (NIR) fluorophore IRDye800CW 15,22.

Since external factors such as ambient light, angle of imaging and distance from tissue to imaging device all may influence imaging results severely 23,24, it is hypothesized that evaluation of the excised specimen might be interesting, with encouraging results reported previously using panitumumab-800CW 25,26. However, although tissue absorption and scattering are minimized in the NIR spectrum (700-900 nm), these phenomena affect fluorescence imaging resulting in higher signals in highly scattering tissue or lower signals in areas that contain high concentrations of blood or in crusts of necrotic tissue for example.

To date, correction for these factors is lacking for FGI in HNC surgery, which might cause different interpretation of fluorescence results within and between patients and centers. In order to tackle the aforementioned technical issues, we applied multi- diameter single-fiber reflectance and single-fiber fluorescence (MDSFR/SFF) spectroscopy to correct for tissue optical properties in order to determine the quantitative intrinsic fluorescence values 23,27-30. The design of our study was adapted based on earlier reports that a pre-dose of unlabeled cetuximab improved cetuximab-800CW FGI results, most likely due to increased off-target receptor occupancy and tissue sequestration 31. However, to date administration of a single flat-dose of cetuximab-800CW has not been compared to administration of cetuximab- 800CW preceded by an unlabeled dose of cetuximab, nor has quantification of intrinsic fluorescence values been included in dose-escalating studies to find the optimal cetuximab-800CW dose for FGI.

In this study, we determined the safety and optimal dosing regimen of cetuximab-800CW for FGI through *real-time* intraoperative fluorescence visualization and quantification of intrinsic fluorescence values and evaluated the clinical added value of back-table FGI for resection margin assessment.

## Methods

### Clinical Trial design

This phase-I safety, feasibility and dose- escalation study was performed at the department of Oral & Maxillofacial Surgery of the University Medical Center Groningen (UMCG, Groningen, The Netherlands). Fifteen patients with histopathological proven HNSCC that were scheduled for tumor resection were included. Patients were identified after the HNC multidisciplinary tumor board agreed on surgery as the primary treatment modality. Written and signed informed consent was obtained prior to any study-related procedure. The study was approved by the Institutional Review Board (IRB) of the UMCG (METc 2016/395) and conducted according to the Dutch Act on Medical Research involving Human Subjects (WMO) and the principles of the Declaration of Helsinki (adapted version Fortaleza, Brazil, 2013). The trial was registered at www.clinicaltrials.gov (NCT03134846).

**Figure 1:**
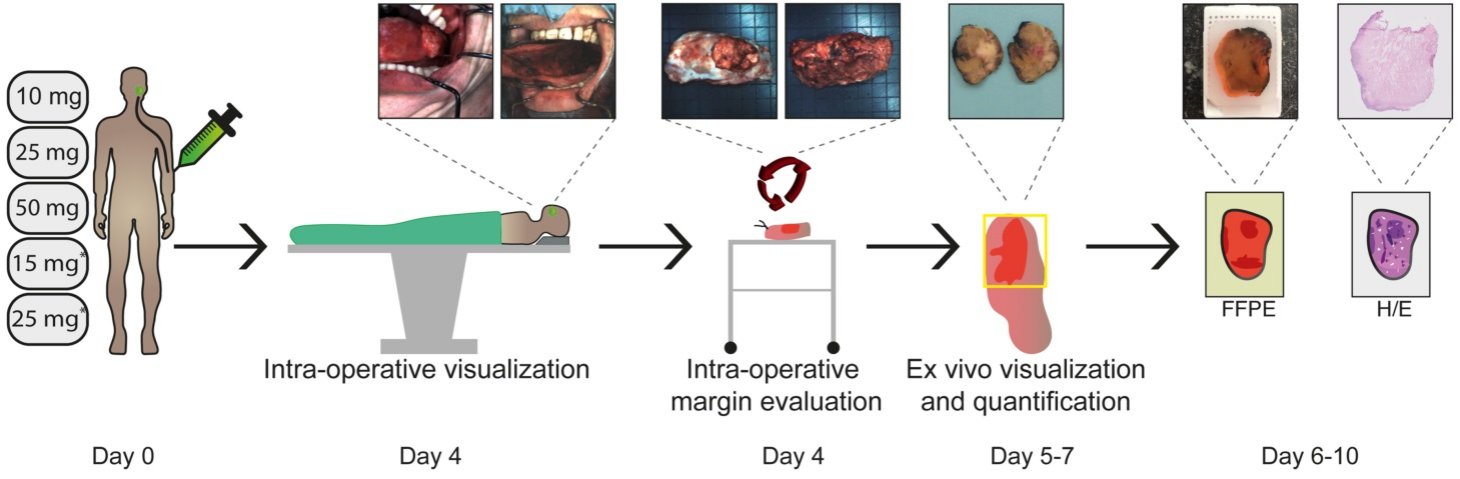
** Study workflow.** Five cohorts of three HNC patients received cetuximab-800CW systemically: three single dose cohorts (10, 25, 50 mg) and two cohorts pre-dosed with 75 mg unlabeled cetuximab (15 or 25 mg). Fluorescence visualization was performed before and after excision of the tumor *in vivo*. Subsequently, back-table fluorescence-guided imaging of the fresh surgical specimen was performed to evaluate the resection margin status. Visualization and quantification of fluorescence was performed during all subsequent steps of standard histopathological processing and correlated to histopathology. Abbreviations: FFPE: Formalin-Fixed Paraffin Embedded, H/E: Hematoxylin and Eosin. *75 mg unlabeled cetuximab is administered one hour prior to cetuximab-800CW administration.

We performed an adapted phase-I dose- escalation study (5x3 design) adhering to the FDA guidelines (Guidance for Industry, Developing Medical Imaging Drug and Biological Products, Part 2 Clinical Indications). All fifteen patients received cetuximab-800CW four days prior to surgery. The dose-escalation study was divided into two parts. Part I involved a single intravenous (IV) administration of cetuximab-800CW (10, 25 or 50 mg single-dose). After an interim analysis, part II commenced, in which patients received 75mg unlabeled cetuximab IV 1h prior to IV administration of cetuximab-800CW (15 or 25 mg). This 1 h interval was based on previous studies on pre-dosing with cetuximab 15,31. The study workflow is depicted in Figure 1.

### GMP synthesis of cetuximab-800CW

Clinical grade cetuximab-800CW (peak excitation and emission wavelength of 778 and 795 nm respectively) was produced in the Good Manufacturing Practice (GMP) facility of the UMCG and released by a certified Qualified Person (QP). A detailed description of the production process has been described previously 32. Briefly, commercially available cetuximab (Erbitux®) 5 mg/mL was conjugated to the near-infrared fluorescence dye IRDye800CW (LI-COR Biosciences, Lincoln, NE, USA) and purified using PD-10 buffer exchange columns (GE Healthcare, Chicago, IL, USA). Cetuximab- 800CW was formulated in a sodium-phosphate buffer at a concentration of 1 mg/mL. All study patients received cetuximab-800CW through a single IV bolus injection.

### Safety measurements

Patients underwent a medical screening procedure before enrollment in the study, consisting of measurements of vital signs, a 12-lead electrocardiogram (ECG) and laboratory tests (including a serum pregnancy test for woman of childbearing potential). Once enrolled, an ECG was performed 1 h after administration of cetuximab-800CW. Vital signs were measured before and after tracer administration (directly and after 1 h). Patients were asked for signs and symptoms before and after tracer administration (directly and after 1 h). After surgery, standard postoperative (outpatient clinic) visits were performed within two weeks. Adverse event assessment was performed according to the National Cancer Institute Common Terminology Criteria for Adverse Events (CTCAE) version 4.0. Serious adverse events (SAE), if present, were reported to the IRB, the independent Data Safety Monitoring Board (DSMB) and the Dutch central committee on research involving human subjects (CCMO). SAE's were followed up until fully resolved or a stable medical situation was achieved.

### Surgical procedure (standard of care)

All patients underwent tumor resection according to standard surgery protocols. Depending on preoperative TNM staging, a sentinel lymph node biopsy (lymph node mapping using the peritumoral administration of Technetium-99m-labelled nanocolloid one day prior to surgery and subsequent intraoperative detection using a gamma-probe) or a lymph node dissection was performed. Based on our previous experience in the UMCG with application of FGI, there was minimal interference with standard of care 13,16,18.

### *In vivo* fluorescence imaging

Surgical procedures were performed according to standard of care and were prioritized over any study-related procedures. Visualization of fluorescence was performed prior to incision of the primary tumor and directly after excision of the surgical wound bed. During fluorescence imaging, the ambient light in the surgical theatre was dimmed to minimize influence on fluorescence imaging results. The use of methylene blue in sentinel node procedures was avoided. The use of fluorescent skin markers and (green) fluorescent sterile drapes was minimalized to prevent interference with fluorescence imaging results 18. It should be noted that *in vivo* imaging of the tumor in the oral cavity is only performed to visualize fluorescence and observe fluorescence patterns. All measurements of fluorescence intensities are performed on the excised specimen.

### Fluorescence cameras

Two intraoperative fluorescence imaging devices were used in this study to detect cetuximab-800CW. Depending on the location of the tumor, either a custom build fluorescence endoscopy platform (SurgVision BV., Groningen, The Netherlands) attached to a flexible nasendoscope (Karl Storz, Tuttlingen, Germany), or an intraoperative fluorescence camera system (Explorer Air®, SurgVision BV., Groningen, The Netherlands) was used during surgery. Both systems provide *real-time* simultaneously fluorescence and white light (color) images and videos. Fluorescence is excited by either a NIR laser (fluorescence nasendoscopic system) or light emitting diodes (Explorer Air®), both of which have an excitation peak specific for cetuximab-800CW detection. Filtered white light is used to provide illumination for the color images. A software user interface is provided to allow the user control over camera settings and to display the color and fluorescence images. As output, both snapshots as well as videos can be recorded and stored in TIFF format. The working distance of the imaging system above the surgical field was set at 3-5 cm for the fluorescence nasendoscopic system and 20 cm for the Explorer Air®. All images were initially obtained with a fluorescence exposure time of 50 ms and a set gain of 300, which could be adjusted depending on fluorescence imaging results.

### Back-table FGI and specimen processing

Immediately after excision, the surgical specimen was marked using sutures for orientation and cross-correlation with histopathology. Subsequently, back-table FGI was performed of all resection planes of the fresh surgical specimen*.* Two back-table FGI devices were used in parallel, the Explorer Air® coupled to a closed-field imaging box (Vault®, SurgVision BV., Groningen, The Netherlands) and a PEARL-trilogy ® imager with a larger adapted sample stage (LI-COR BioSciences Inc., Lincoln, NE, USA). The Pearl-trilogy® detects fluorescence using a CCD camera in the NIR wavelength (peak excitation 785 nm, peak emission 820 nm). The field of view of 11.2 cm x 8.4 cm and the focus point can be adjusted based on specimen height. Fluorescence was scored at the resection margin by physicians blinded for histology results and scored positive if the TBR was higher than two (the specimen was used as its own internal control).

After back-table FGI, the specimen was formalin fixed for at least 24 h, depending on specimen size, after which the specimen was inked to mark resection margins on final histopathology with black and blue ink.

The formalin-fixed surgical specimen was serially sliced into approximately 0.4 cm thick tissue slices. White-light images were acquired during and directly after tissue slicing for orientation purposes. After slicing, fluorescence imaging was performed on both sides of each tissue slice using the PEARL- trilogy®. Subsequently, a pathologist blinded for the fluorescence images selected regions of interest based on gross examination by visual inspection and palpation, which were further embedded in paraffin blocks for standard histopathological analysis. In case of the presence of bone, tissue was decalcified before further processing. A standardized workflow was used in order to cross-correlate final histopathology results with recorded fluorescence images of tissue slices of interest 18. Subsequently, hematoxylin and eosin (H/E) staining was performed on 4 μm tissue sections for histopathological analysis and analyzed by a board-certified pathologist blinded for fluorescence imaging results. A tumor-positive resection margin was defined as viable tumor tissue within 1 mm of the resection margin according to European guidelines in HNSCC treatment 33.

### *Ex vivo* MSDFR/SFF spectroscopy

The MDSFR/SFF spectroscopy device acquires two reflectance spectra by using two different optical fibers and subsequently one raw fluorescence spectrum, as described previously 18. Briefly, the scattering and absorption coefficients are determined from the two reflectance spectra. These are then used to calculate the intrinsic fluorescence values (*Q·μ*^f^_a,x_) of cetuximab-800CW, by correcting the raw fluorescence spectrum for the calculated tissue optical properties 34. Intrinsic fluorescence values (*Q·μ*^f^_a,x_) of cetuximab-800CW are defined as a product of the quantum efficiency across the emission spectrum, *Q*[-], where *Q* is the fluorescence quantum yield of IRDye-800CW and μ_af_ [mm^-1^] is the tracer absorption coefficient at the excitation wavelength. The data is collected using direct contact measurements. All measurements were repeated in triplicate and median values were calculated per measurement location.

We calculated the concentration of IRDye800CW based on an assumed extinction coefficient (31120.4 mm^-1^), fluorescence quantum yield (0.09) and the cetuximab-800CW load factor (1:2) for each cohort 12 *ex vivo* in the tissue specimens.

### *Ex vivo* validation of fluorescence intensities and localization

PEARL-trilogy® images of tissue slices that contained tumor tissue and non-tumor tissue were used to calculate the mean fluorescence intensity (MFI) of both tumor and non-tumor tissue.

A board-certified pathologist, blinded from the fluorescence images, drew region of interests (ROI) of areas that contained tumor on 4 μm H/E slides and subsequently overlaid on the corresponding tissue slice. MFIs were calculated based on these ROIs on the tissue slices of both tumor tissue and non-tumor tissue (i.e. all tissue in the tissue slice diagnosed as non-tumor tissue). A tumor-to-background-ratio (TBR) was calculated by dividing tumor ROI (MFI tumor) with the background ROI (MFI non-tumor tissue) per tissue slice. Median TBR values were calculated on a per patient base.

Immunohistochemical analysis was performed on 4 μm thick tissue sections which contained tumor and non-tumor tissue to correlate fluorescence intensities with EGFR expression (VENTANA Benchmark Ultra anti-EGFR (Clone 3C6), Roche, Basel, Switzerland). The EGFR-membrane expression was evaluated following a pre-defined scoring system (0, +, ++ or +++) by a board-certified pathologist.

An inverted microscope (DMI6000B, Leica Biosystems GmbH, Wetzlar, Germany) was used for fluorescence microscopy with a pixel size of 6.45 µm and a field of view of 120 × 120 mm. To optimize NIR visualization, the microscope was equipped with a NIR LED light source ranging up to 900 nm (X-Cite 200DC, Excelitas Technologies, Waltham, MA, USA), a NIR filter set (microscope two band- pass filters 850-890 m-2p and a long-pass emission filter HQ800795LP; Chroma Technology Corp, Bellows Falls, VT, USA) and a monochrome DFC365 FX fluorescence camera (1.4 M Pixel CCD, Leica Biosystems GmbH). An acquisition time of 12 s was used for images of the 800 nm channel.

### Cetuximab-800CW tracer integrity

Sodium dodecyl sulfate polyacrylamide gel electrophoresis (SDS-PAGE) was performed both on blood samples of the included patients collected on day four and on lysate of fresh frozen tumor tissue to ensure intactness of the cetuximab-800CW conjugate, as previously described (ProSieve® Quadcolor™ Protein Markers, Lonza Rockland Inc., ME, USA and Mini-Protean TGX Precast Protein Gel, Bio-Rad Laboratories Inc., California, USA) 18,35. Results were compared with labeled and unlabeled cetuximab. The gel was scanned with the Odyssey CLX® flatbed scanner (LI-COR Biosciences Inc. Lincoln, NE, USA) at the 800 nm channel.

### Statistical analysis

Descriptive statistics were performed on the patient demographics. MFI was calculated using ImageJ Fiji (version 2.0.0-rc-68/1.52h) as total counts per ROI pixel area in both tumor and non-tumor tissue. Data was tested for Gaussian distribution using Anderson-Darling and Shapiro-Wilk test; none of the data was normally distributed. Differences between paired and unpaired data was tested using a Wilcoxon test and Mann-Whitney test respectively. Data is presented as median values with interquartile ranges. For statistical analysis and graph design, GraphPad Prism (version 8.0, GraphPad Software Inc, San Diego, California, USA) was used.

## Results

Fifteen patients with HNSCC were included in this study. The age of the patients ranged from 48 to 86 years. Administration of cetuximab-800CW was considered to be well tolerated in all dose-cohorts, with five possibly related adverse events throughout the study that were all limited to CTCAE grade I events (Table 1 and Table S1). The grade II event (i.e. bronchospasm) was considered not to be imaging agent related but iatrogenic since the administration speed was accidently set 10x higher than recommended.

### Evaluation of resection margin status

**Figure 2:**
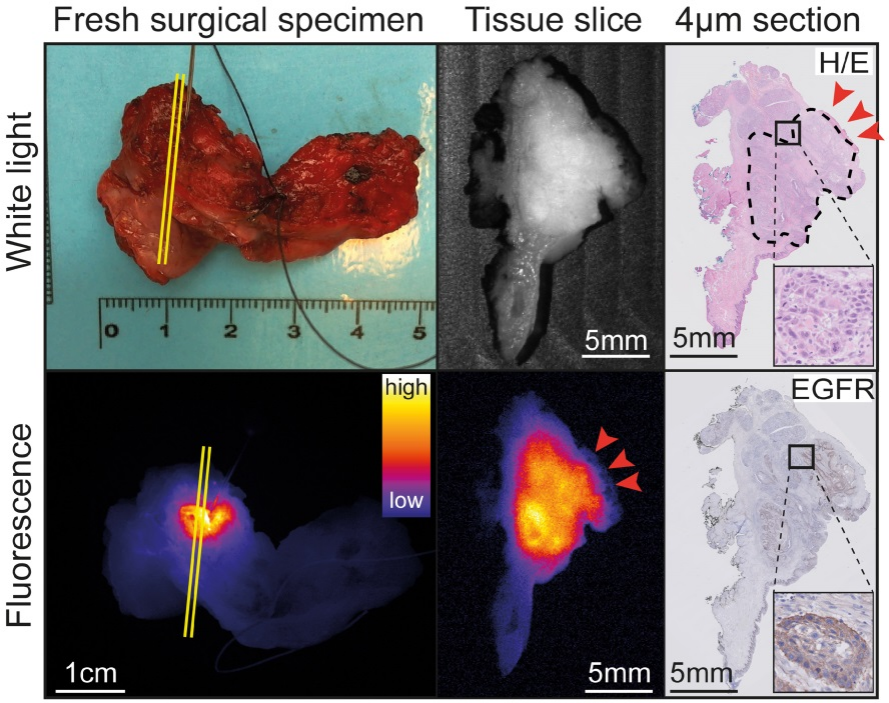
** Representative example of back-table FGI of a tumor-positive resection margin.** Back-table fluorescence-guided imaging (FGI) of a fresh surgical specimen, with a clearly localized increased fluorescent lesion, indicating a tumor-positive ventral resection margin of a tumor located on the floor of the mouth upon final histopathology. The tumor-positive resection margin on the tissue slice and section (red arrows) correspond to the location on the fresh surgical specimen (yellow lines). Abbreviations: H/E: Hematoxylin and Eosin. EGFR: Endothelial Growth Factor Receptor.

Four patients were diagnosed with a tumor-positive resection margin (i.e. <1 mm) based on standard histopathology (Table 1). During back-table FGI of the fresh surgical specimens, all four tumor-positive resection margins were correctly identified (sensitivity 100%; Figure 2 and Table 2). *In vivo* visualization of the wound bed did not show remaining fluorescent lesions. This was in line with final histopathology, since all four tumor-positive resection margins were based on the presence of viable tumor cells within 0.3-1 mm of the resection margin, suggesting there was no residual tumor in the wound bed.

Eleven out of fifteen patients were diagnosed with a tumor-negative resection margin upon final histopathology (Table 1). Back-table FGI of the fresh surgical specimens showed no fluorescent lesions on the resection margins in 10/11 patients (specificity 91%; Table 2). The remaining resection margin (2 mm) was evaluated as tumor-positive based on a fluorescent lesion (Figure S1).

**Figure 3:**
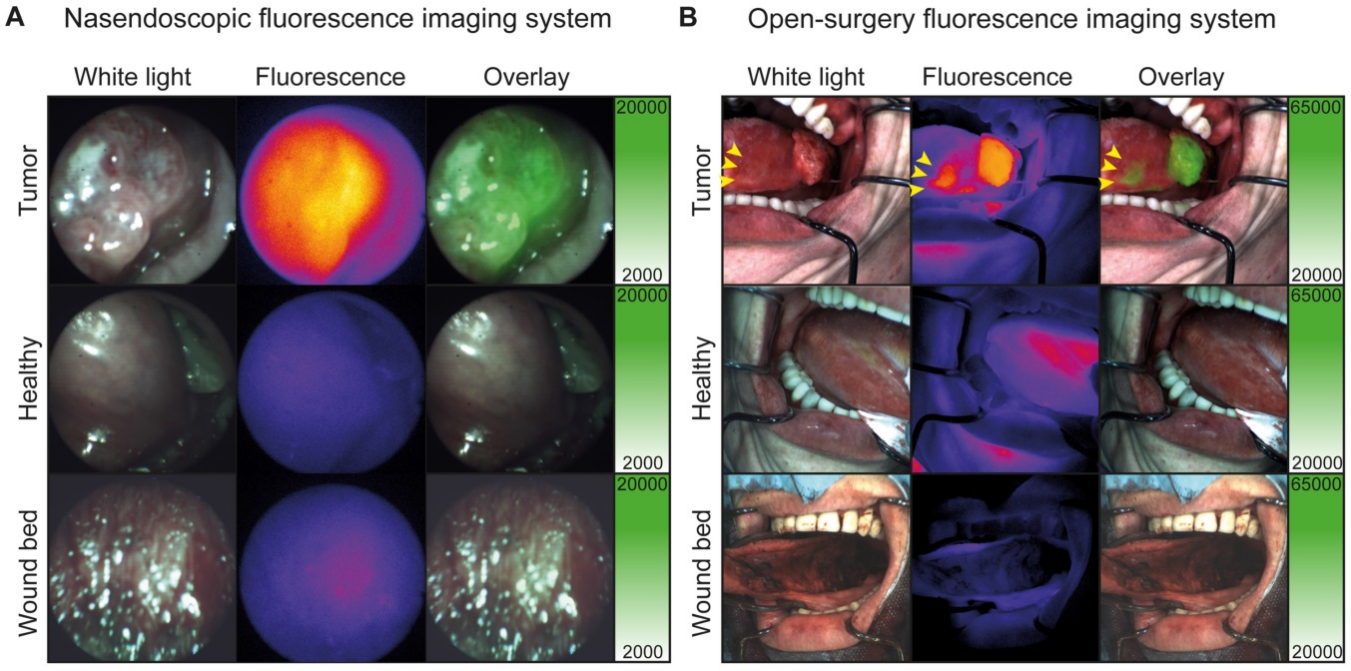
***In vivo* fluorescence visualization during surgery.** A: Representative fluorescence imaging using the nasendoscopic fluorescence imaging system of a buccal HNSCC tumor. B: Representative fluorescence imaging using Explorer Air® fluorescence imaging system of a HNSCC tumor of the tongue. Yellow arrows indicate a separate second tumor lesion diagnosed using cetuximab-800CW fluorescence.

In addition, one satellite tumor lesion was detected during *in vivo* FGI, which was located anterior of the main tumor lesion (Figure 3B, yellow arrows), highlighting the potential added value of *real-time* intraoperative FGI. Fluorescence imaging procedures prolonged the standard procedure with approximately 10 min and did not interfere with the standard of care. Difficult and easily accessible tumors and surgical cavities could be visualized *in vivo* using the flexible nasendoscopic camera and the Explorer Air® respectively (Figure 3A-B and Video S1).

### Visualization and quantification of cetuximab-800CW fluorescence

**Figure 4:**
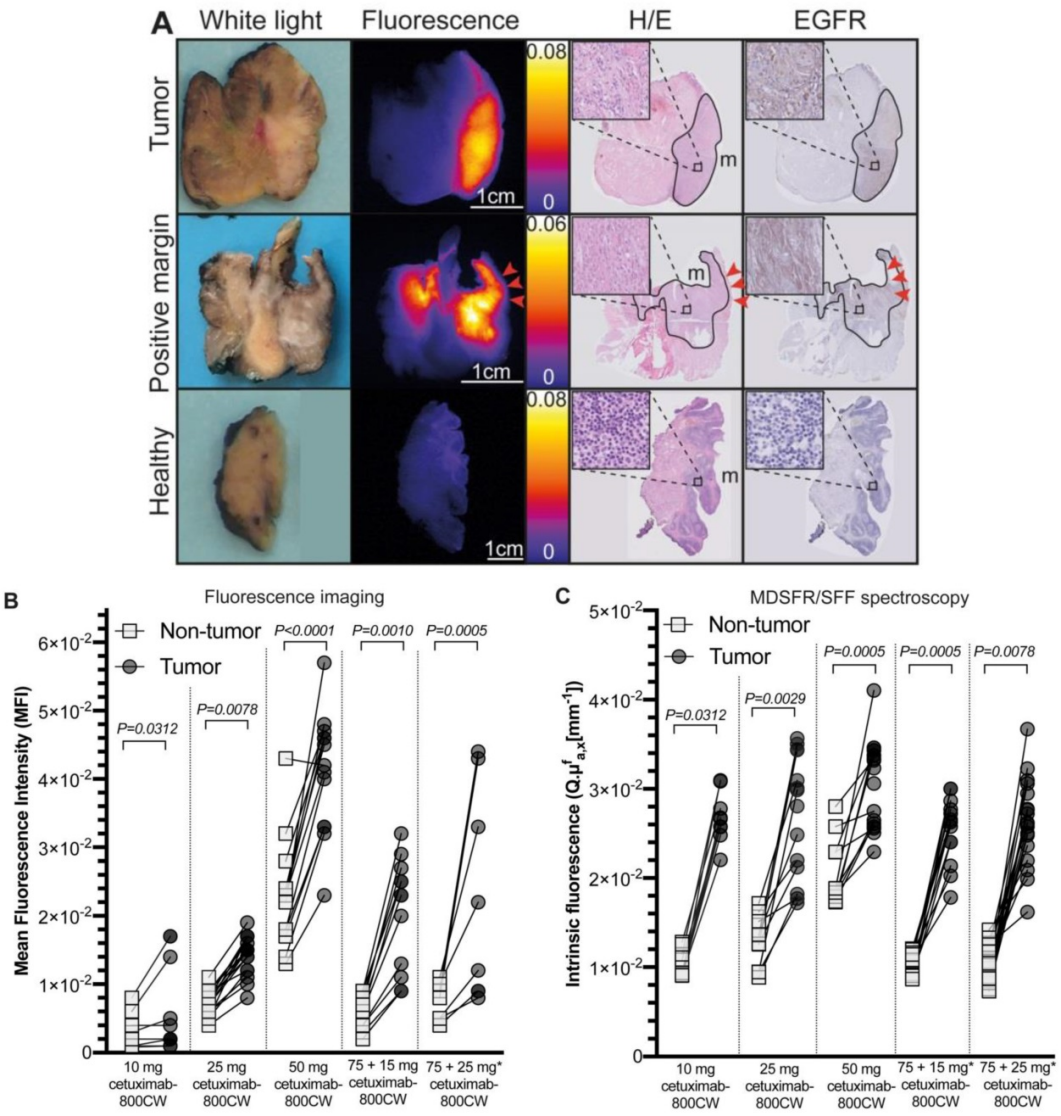
***Ex vivo* correlation and quantification of cetuximab-800CW fluorescence.** A: Tumor tissue is delineated on Hematoxylin and Eosin (H/E) staining, which correlates to the fluorescence imaging results on the tissue slices and EGFR expression on tissue slice containing tumor (upper row) and a tumor-positive resection margin (middle row), whereas a non-tumor tissue slices shows low fluorescence intensities and no EGFR expression (lower row). Red arrows indicate a tumor-positive margin of 0.3 mm. B: Tumor tissue showed significantly increased fluorescence intensities compared to non-tumor tissue on fluorescence images of tissue slices. C: Intrinsic fluorescence values were significantly higher in tumor tissue compared to non-tumor tissue. * 75 mg cetuximab is administered one hour prior to cetuximab-800CW administration. Dots represent median intrinsic fluorescence values per measurements location. Error bars represent median values and interquartile range. Abbreviations: H/E: Hematoxylin and Eosin. EGFR: Endothelial Growth Factor Receptor.

To objectivate the potential of cetuximab-800CW for the discrimination of tumor tissue versus non-tumor tissue, we first calculated the tumor fluorescence of the ROI of tissue slices (N=61). Here, significantly higher fluorescence intensities in tumor tissue compared to non-tumor tissue were detected for all cohorts (TBR 10 mg: 1.61±0.93, *p=0.0312*; 25 mg: 2.02±0.55, *p=0.0078*; 50 mg: 1.81±0.32, *p<0.0001*; 75 + 15 mg: 3.06±0.43, *p=0.0010*; 75 + 25 mg: 3.10±2.53, *p=0.0005*; Figure 4A-B and Table 3), with increasing TBRs in the pre-dosed cohorts. To further validate these findings, quantification of the intrinsic fluorescence values by MDSFR/SFF on tissue slices (N=46) confirmed these findings in all dose-cohorts (TBRs 10 mg: 2.86±0.29, *p=0.0312*; 25 mg: 1.99±1.62, *p=0.0078*; 50 mg: 1.48±0.52, *p<0.0001*; 75 + 15 mg: 2.50±0.19, *p=0.0010*; 75 + 25 mg: 2.81±1.27, *p=0.0005*; Figure 4C and Table 3).

A dose-dependent increase in background fluorescence in the single-dose cohorts was observed using both fluorescence intensities of the tissue ROI of the fluorescence images (10 mg vs. 25 mg: *p=0.0006,* 25 mg vs. 50 mg:* p<0.0001*) and quantification of intrinsic fluorescence values (10 mg vs. 25 mg: *p<0.0001*, 25 mg vs. 50 mg: *p<0.0001*), whereas this remained consistently low in the cohorts pre-dosed with unlabeled cetuximab, without a significant difference between both cohorts (*p=0.4320* and *p=0.6808* for fluorescence imaging and MDSFR/SFF spectroscopy respectively). Median intrinsic fluorescence values in non-tumor tissue were significantly lower in the 75 + 25 mg cohort compared to the 25 mg single-dose cohort (0.0101 mm^-1^ vs. 0.0131 mm^-1^, *p=0.0096*, respectively).

Fluorescence intensities of tumor tissue increased with increasing doses in the single dose cohorts (10 mg vs. 25 mg: *p=0.0044,* 25 mg vs. 50 mg:* p<0.0001*), but not in the in the pre-dose cohorts (75 + 15 mg vs. 75 + 25 mg: *p=0.8959)* using tissue fluorescence of the ROI of tissue slices. Quantification of intrinsic fluorescence values by MDSFR/SFF spectroscopy showed no dose-dependent increase (10 mg vs. 25 mg: *p=0.8291,* 25 mg vs. 50 mg:* p=0.2351,* 75 + 15 mg vs. 75 + 25mg: *p=0.7573).*

After correcting *ex vivo* fluorescence measurements obtained by MDSFR/SFF spectroscopy for tissue optical properties, the intrinsic fluorescence values (i.e. corrected) decreased in the majority of patients compared to the uncorrected values. Scattering and absorption coefficients mainly affected non-tumor tissue measurements (Figure S2), resulting in higher, although not significant, TBRs when comparing MDSFR/SFF spectroscopy TBRs to fluorescence imaging TBRs (2.50 ± 1.00vs. 2.13 ± 1.04 respectively; *p=0.9780*, Figure S3).

**Figure 5:**
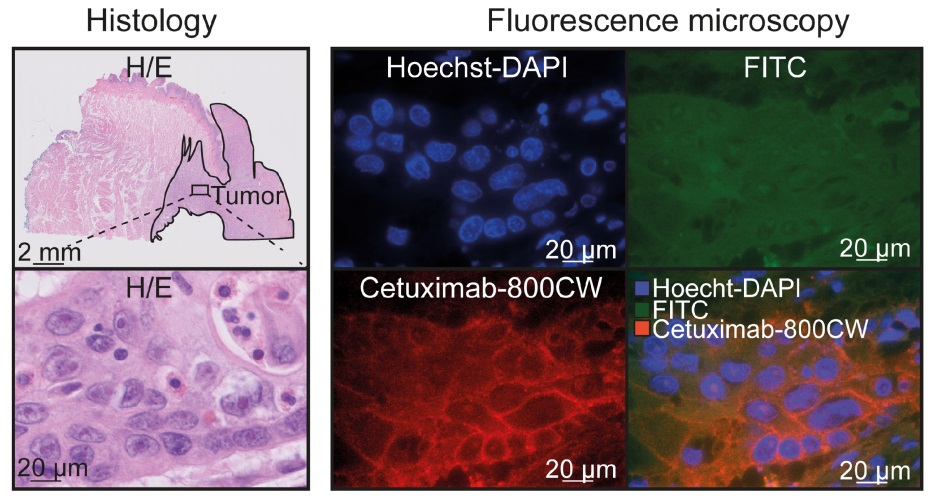
** Cetuximab-800CW binding specificity.** Cetuximab-800CW fluorescence was located around the tumor cells, indicating specific binding. A Hoechst-DAPI staining was performed to visualize cell nuclei and the FITC channel was used to discriminate between autofluorescence and cetuximab-800CW derived fluorescence on the 800 nm channel. Abbreviations: H/E: Hematoxylin and Eosin. FITC: Fluorescein isothiocyanate.

EGFR immunohistochemistry showed expression in 96% of the tumors, in contrast to deeper seated non-tumor tissue (e.g. fat, connective tissue and muscle tissue; N=27 tissue sections; Figure S4). In addition, nineteen tissue sections contained salivary glands, of which nine (47%) showed a moderate EGFR expression. To further validate cetuximab-800CW binding specificity, fluorescence microscopy showed cetuximab-800CW was localized around tumor cell membranes (Figure 5).

### Cetuximab-800CW integrity

Cetuximab-800CW integrity was confirmed four days after administration in blood samples and tumor lysates of fresh surgical specimen samples (Figure S5).

## Discussion

In this study, the optimal dose of cetuximab-800CW appeared to be a dose of 15 mg cetuximab-800CW preceded by 75 mg of unlabeled cetuximab. Further increasing the cetuximab-800CW dose after pre-dosing did not significantly increase the tumor-to-background ratios.

In fluorescence guided imaging, evaluation of the resection margin status is most reliably done *ex vivo*, as previous studies of our group and others show 18,25,36. In addition, determining the optimal dose *ex vivo* can be done more reliable since* in vivo* imaging does not allow collection of data in a standardized manner which can influence results 23. In the current study, we used *ex vivo* tissue fluorescence intensities of the tissue slices of tumor and adjacent non-tumor tissue to calculate the TBR. We confirmed these outcomes with MDSFR/SFF spectroscopy by correcting for tissue optical properties, although obtaining visual information from the fluorescence images is considered to be most clinically relevant for the for the intraoperative evaluation of the margin status since it provides wide-field information.

Here, we studied single-dose cetuximab-800CW cohorts as well as cohorts pre-dosed with an unlabeled dose cetuximab followed by cetuximab- 800CW. Although visualization of fluorescence was possible even in the lower single-doses cohorts, a clinically relevant discrimination between tumor and non-tumor tissue was obtained in the pre-dosed cohorts (Figure S6). Here, background fluorescence remained consistently low, while a dose-dependent increase was observed with in the single-dose cohorts, indicating off-target receptor saturation when pre-dosing.

A previous study using 25 mg/m^2^ cetuximab- 800CW showed that pre-dosing with 100 mg unlabeled cetuximab improves tumor visualization over 10 mg unlabeled cetuximab 31. Another study using panitumumab-800CW showed no difference between pre-dosed and single dose cohorts 37. In the current study, we used a two-to-threefold lower cetuximab-800CW dose than previously described, which might decrease the risk of toxicity and costs. Although it is likely that the total dose of cetuximab (i.e. 90 mg in the optimal dose cohort) is relevant for potential toxicities, this is still considerably lower than previously described 15,31. Importantly, the lower dose of cetuximab-800CW did not influence our imaging results, because we used a highly sensitive fluorescence camera system optimized for IRDye800CW visualization, whereas Rosenthal et al. used a system optimized for Indocyanine Green visualization (peak absorption and emission 806 nm and 830 nm respectively) 31.

In addition, this study demonstrates the added value of quantification of intrinsic fluorescence values through correcting for tissue scattering and absorption coefficients for verifying the optimal dose. When interpreting fluorescence imaging results, one should realize that, regardless of the biodistribution of a fluorescent tracer, fluorescence intensities are influenced by several factors, such as the tissue optical properties, ambient light, imaging distance and the sensitivity and dynamic range of the fluorescence camera 23,29,38,39. Previous studies investigating EGFR-targeted imaging in HNC surgery solely used fluorescence visualization to report outcome parameters 15,22, although recently techniques have been described to quantify fluorescence 40. Here, we demonstrate the added value of MDSFR/SFF spectroscopy in dose-finding studies, as we show the uncorrected fluorescence results differ significantly from corrected fluorescence values.

The preliminary sensitivity and specificity of 100% and 91% respectively clearly demonstrate the potential added value of back-table FGI of the resection specimen for intraoperative clinical decision making, despite our limited patient sample. All tumor-positive resection margins had initially been missed by visual and tactile inspection. In the current study which involves fifteen patients, in four cases a tumor-positive resection margin was detected (4/15, 27%). There was no particular tumor-phenotypic feature that contributed to the missed positive margins. In fact, all tumors were moderately differentiated squamous cell carcinomas ranging from T1 to T4 stages. Although we observed one false-positive fluorescent lesion on the resection margin, a high sensitivity is crucial in HNC treatment, as leaving residual tumor negatively influences treatment outcome and survival 4. Back-table FGI serves as a 'red-flag' imaging technique that allows the surgeon to perform more targeted fresh frozen section analysis or to perform a re-resection while the patient is still anesthetized. This potentially prevents second surgery or (extensive) adjuvant treatment regimens. This is congruent with data from previous studies investigating the added value of back-table FGI 16,18. The benefit of a specimen-driven approach for margin evaluation is that it can be performed in a controlled and standardized environment, which overcomes previously described factors that influence fluorescence imaging results 23.

In the current study, we showed that it was feasible to perform FGI using a flexible nasendoscope which allowed imaging of locations that were difficult to assess using an open-surgery fluorescence camera. Although the fiber optic approach reduced imaging resolution and detection sensitivity compared to the wide-field fluorescence imaging system, we believe the nasendoscopic system can assist in detecting undisclosed lesions in the upper aerodigestive tract.

EGFR was validated as a target using immunohistochemistry. Although we observed a heterogeneous EGFR expression in tumor tissue microscopically, this did not influence macroscopic fluorescence imaging results. In the current study, 96% of HNSCC tissue sections expressed EGFR. Although the normal mucosa also expressed EGFR, this was not observed in the muscle, fat and connective tissue that forms the basal resection margin, where 80% of tumor-positive resection margins are located 9. Although we observed EGFR expression in salivary glands which is in line with literature, this did no influence the evaluation of resection margins in the current study.

In conclusion, we found that a dose of 75 mg unlabeled cetuximab followed by 15 mg cetuximab-800CW was well tolerated and allowed optimal discrimination between tumor and non-tumor tissue. MDSFR/SFF spectroscopy verifies the localization and quantifies the concentration of cetuximab-800CW in tumor and non-tumor tissue by correcting for tissue optical properties and provides important information on the tracer biodistribution, which supports our rationale for the optimal dose. The results of this study have led to the initiation of a currently ongoing phase-II clinical study in our institute (NCT03134846), investigating the clinical value of back-table FGI using cetuximab-800CW in a larger population of HNSCC patients.

## Supplementary Material

Supplementary figures, tables, and video legend.

Supplementary video 1.

## Figures and Tables

**Table 1:** Patient and surgical specimen characteristics.

Patient characteristics	10 mg CTX-800CW N=3	25 mg CTX-800CW N=3	50 mg CTX-800CW N=3	75 mg CTX + 15 mg CTX-800CW N=3	75 mg CTX + 25 mg CTX-800CW N=3
**Age (mean years, range)**	68 (59-73)	62 (56-71)	63 (48-86)	63 (50-78)	55 (49-64)
**Males (number, percentage)**	2 (53%)	3 (100%)	1 (33%)	1 (33%)	1 (33%)
**Weight (mean kg, range)**	79 (45-104)	77 (57-97)	78 (93-97)	97 (81-140)	82 (58-122)
**Safety**					
Grade I	2	2	0	1	0
Grade II	0	0	0	1	0
**Tumor histology**					
HNSCC	3	3	3	3	3
**Size**					
Max Diameter (mean ±SD mm)	34 (±20)	20 (±7.5)	18 (±7.0)	30 (±11)	26 (±18)
Depth of Invasion (mean ±SD mm)	11 (±3.6)	6 (±1.4)	4 (±0.8)	5 (±3.1)	6 (±5.1)
Surgical margin*					
Tumor-negative	2	2	2	2	3
Tumor-positive	1	1	1	1	0

Patient and surgical specimen characteristics are depicted per cohort. Potential related adverse events are scored according to the Common Terminology Criteria for Adverse Events. *Tumor-negative margin: >5 mm; tumor-positive surgical margin: <1 mm. Abbreviations: CTX: Cetuximab.

**Table 2:** Contingency table of back-table fluorescence-guided imaging results.

	Tumor-positive resection margin	Tumor-negative resection margin	Total
**Back-table FGI positive**	4	1	5
**Back-table FGI negative**	0	10	10
**Total**	4	11	15

All four tumor-positive resection margins were correctly identified using back-table fluorescence-guided imaging (FGI) of the fresh surgical specimens. In addition, ten tumor-negative resection margins were correctly identified. One tumor-negative resection margin was identified as tumor-positive, based on back-table FGI. Abbreviations: FGI: Fluorescence-guided imaging.

**Table 3:** *Ex vivo* tumor-to-background ratio per dose cohort.

Dose-cohort	TBR	
	Visualization	Spectroscopy
10mg	1.61 ± 0.93	2.86 ± 0.29
25mg	2.02 ± 0.55	1.99 ± 1.62
50mg	1.81 ± 0.32	1.48 ± 0.52
15mg*	3.06 ± 0.43	2.50 ± 0.19
25mg*	3.10 ± 2.53	2.81 ± 1.27

Median tumor-to-background ratios shown both for fluorescence visualization using a closed-field imaging device and quantification of intrinsic fluorescence values using MDSFR/SFF spectroscopy.

## Abbreviations

FGI: fluorescence-guided imaging
MDSFR/SFF: multi diameter single fiber reflectance, single fiber fluorescence
HNC: head and neck cancer
HNSCC: head and neck squamous cell carcinoma
EGFR: endothelial growth factor receptor
UMCG: university medical center Groningen
IRB: institutional review board
WMO: Dutch act on medical research involving human subjects
QP: qualified person
IV: intravenous
GMP: good manufacturing practice
ECG: electrocardiogram
CTCAE: national cancer institute common terminology criteria for adverse events
SAE: serious adverse events
DMSB: data monitoring safety board
CCMO: Dutch central committee on research involving human subjects
MFI: mean fluorescence intensity
ROI: region of interest
TBR: tumor-to-background-ratio
SDS-PAGE: sodium dodecyl sulfate polyacrylamide gel electrophoresis
