# Supplementary material for: Fluorescence-guided imaging for resection margin evaluation in head and neck cancer patients using cetuximab-800CW: A quantitative dose-escalation study
Source: Theranostics. 2020 Mar 4;10(9):3994–4005. doi: 10.7150/thno.43227 (PMC7086353; doi:10.7150/thno.43227)
Supplement: Supplementary file 1 — Supplementary figures, tables, and video legend. [file thnov10p3994s1.pdf]

SUPPLEMENTAL TABLES

TABLE S1

	Adverse Event	Grade	Intervention
1	Hypotension (during surgery)	I	I.V. fluid suppletion
2	Temporary QTc prolongation	I	None
3	Temporary QTc prolongation	I	None
4	Flush	I	None
5	Fever	I	None
6	Bronchospasm*	II	2 mg Clemastine I.V. + 4 mg Dexamethosaon IV

Table S1 | Adverse events related to tracer administration

Six possible related adverse occurred due to cetuximab-800CW administration. *The bronchospasm occurred after accidently too rapid infusion (10x higher than planned).

TABLE S2

Dose-cohort	Concentration in tumor *(nmol/mL)*	Concentration in non-tumor tissue *(nmol/mL)*	Ratio
10 mg	4.78 ± 0.36	1.67 ± 0.41	2.86
25 mg	5.45 ± 2.62	2.35 ± 0.77	2.32
50 mg	6.02 ± 1.46	3.37 ± 0.89	1.79
15 mg*	4.50 ± 0.59	1.93 ± 0.22	2.33
25 mg*	4.70 ± 1.22	1.70 ± 0.42	2.81

Table S2 | Cetuximab-800CW concentrations based on MDSFR/SFF spectroscopy measurements in tissue slices.

Increasing single doses of cetuximab-800CW show increasing background fluorescence in non-tumor tissue whereas background fluorescence remains low when an unlabeled pre-dose of cetuximab is administered one hour prior to cetuximab-800CW. Values depicted as median ± IQR. *75 mg cetuximab is administered one hour prior to cetuximab-800CW administration.

SUPPLEMENTAL FIGURES

FIGURE S1

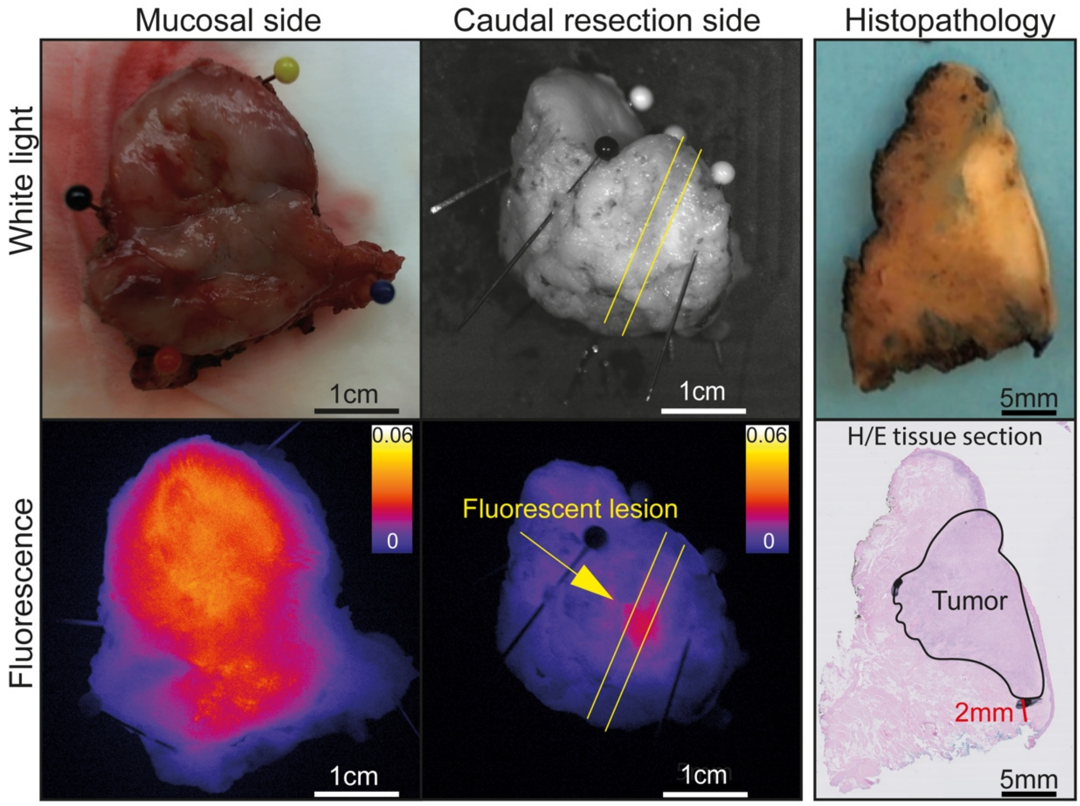

Figure S1 | Back-table fluorescence-guided imaging of a fluorescence-positive but tumor-negative resection margin.

Excised specimen (partial tongue resection) which shows fluorescence at the primary tumor (mucosal side). At the caudal resection margin, a localized fluorescent lesion (yellow arrow) that corresponds to tumor-negative resection margin of 2 mm (caudal resection surface). Tumor is delineated with a solid black line. Abbreviations: H/E: Hematoxylin & Eosin.

FIGURE S2

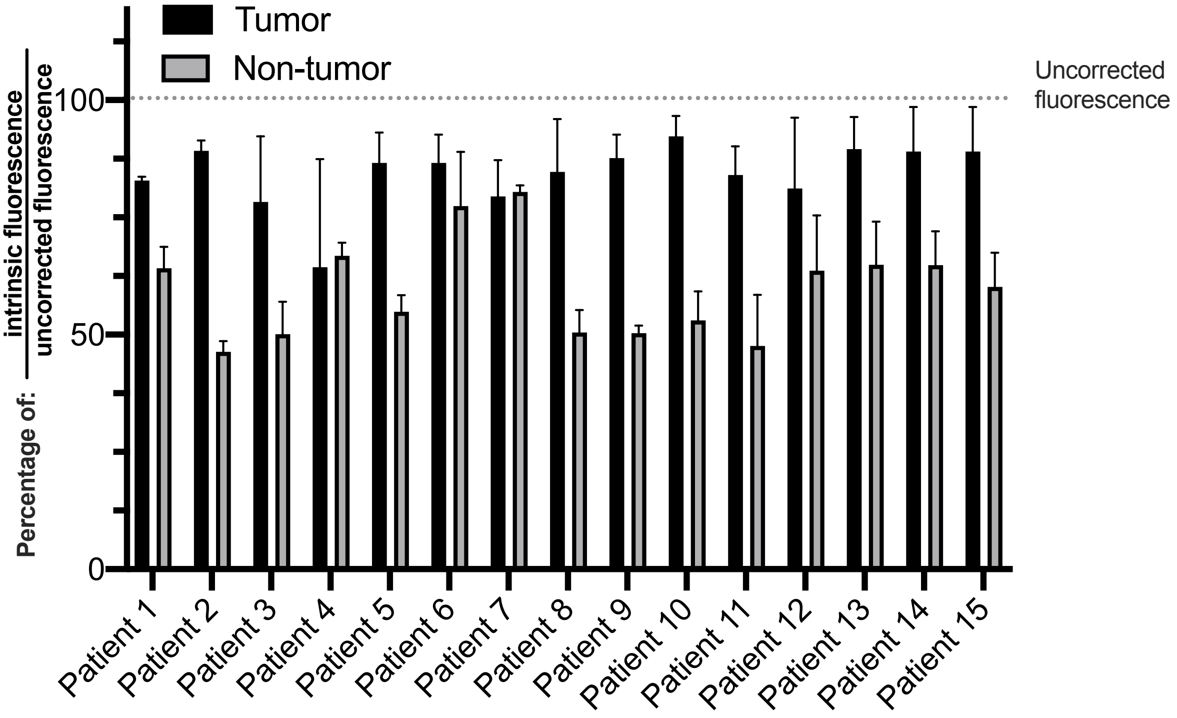

Figure S2 | Influence of tissue scattering and absorption on intrinsic fluorescence values during FGI.

The intrinsic fluorescence values (i.e. corrected for scattering and absorption) are depicted as a percentage of the uncorrected fluorescence values acquired with MDSFR/SFF spectroscopy direct contact measurements. Non-tumor tissue is more affected by tissue optical properties compared to tumor tissue. Bars represent median values with interquartile range.

FIGURE S3

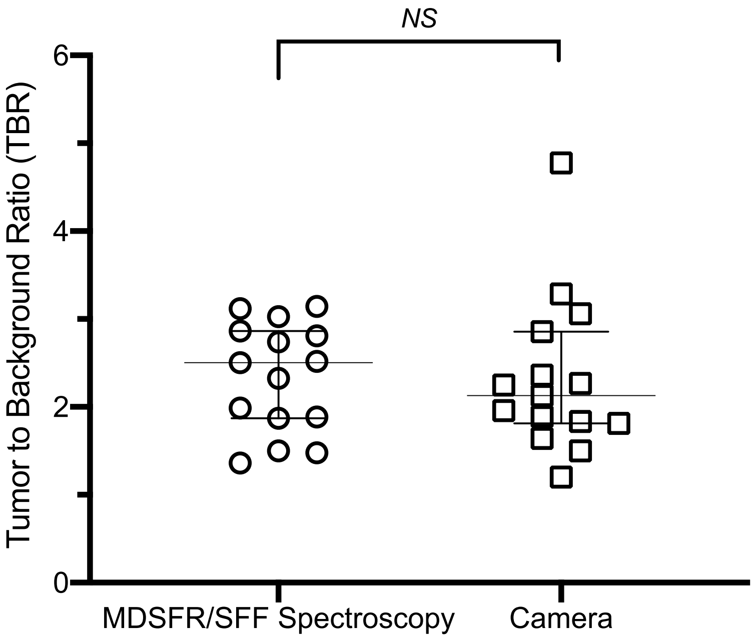

Figure S3 | Difference in TBR between quantification of intrinsic fluorescence values (MDSFR/SFF spectroscopy) versus fluorescence visualization (camera)

Dots represent the median TBRs values of the *in vivo* intrinsic fluorescence values measured using MDSFR/SFF spectroscopy (right) and fluorescence visualization using the fluorescence camera system (left) per patient. Error bars represent median values and interquartile range.

FIGURE S4

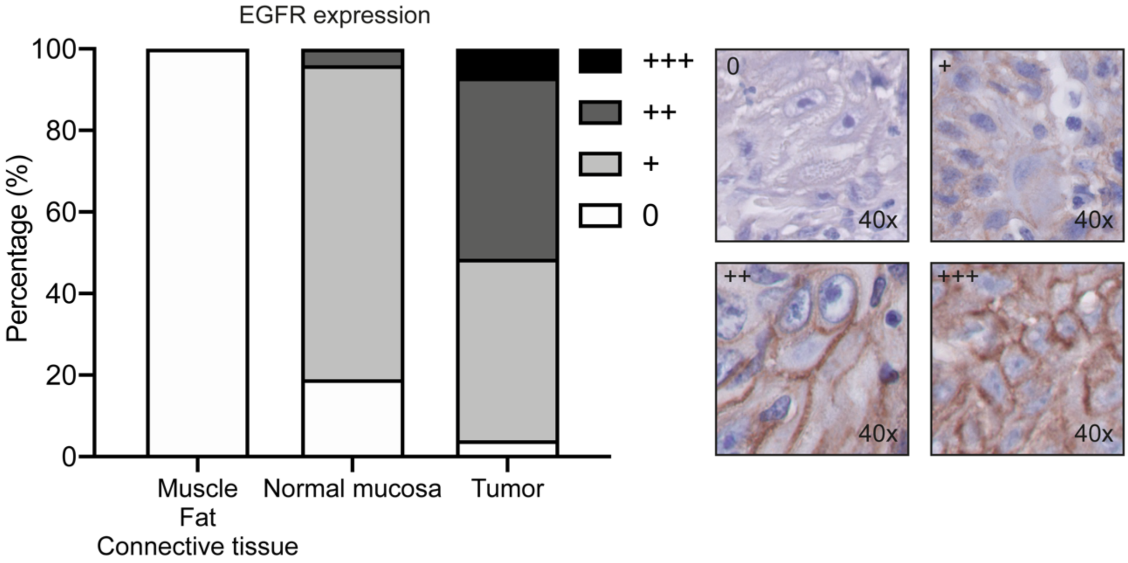

Figure S4 | Endothelial Growth Factor Receptor membrane expression.

Muscle, fat and connective tissue showed no EGFR expression, whereas a clear expression was observed in tumor tissue in 96% of the tissue sections (N=27). Normal mucosa showed in the majority of cases mild (1+) EGFR expression. A representative image of each EGFR staining intensity is depicted.

FIGURE S5

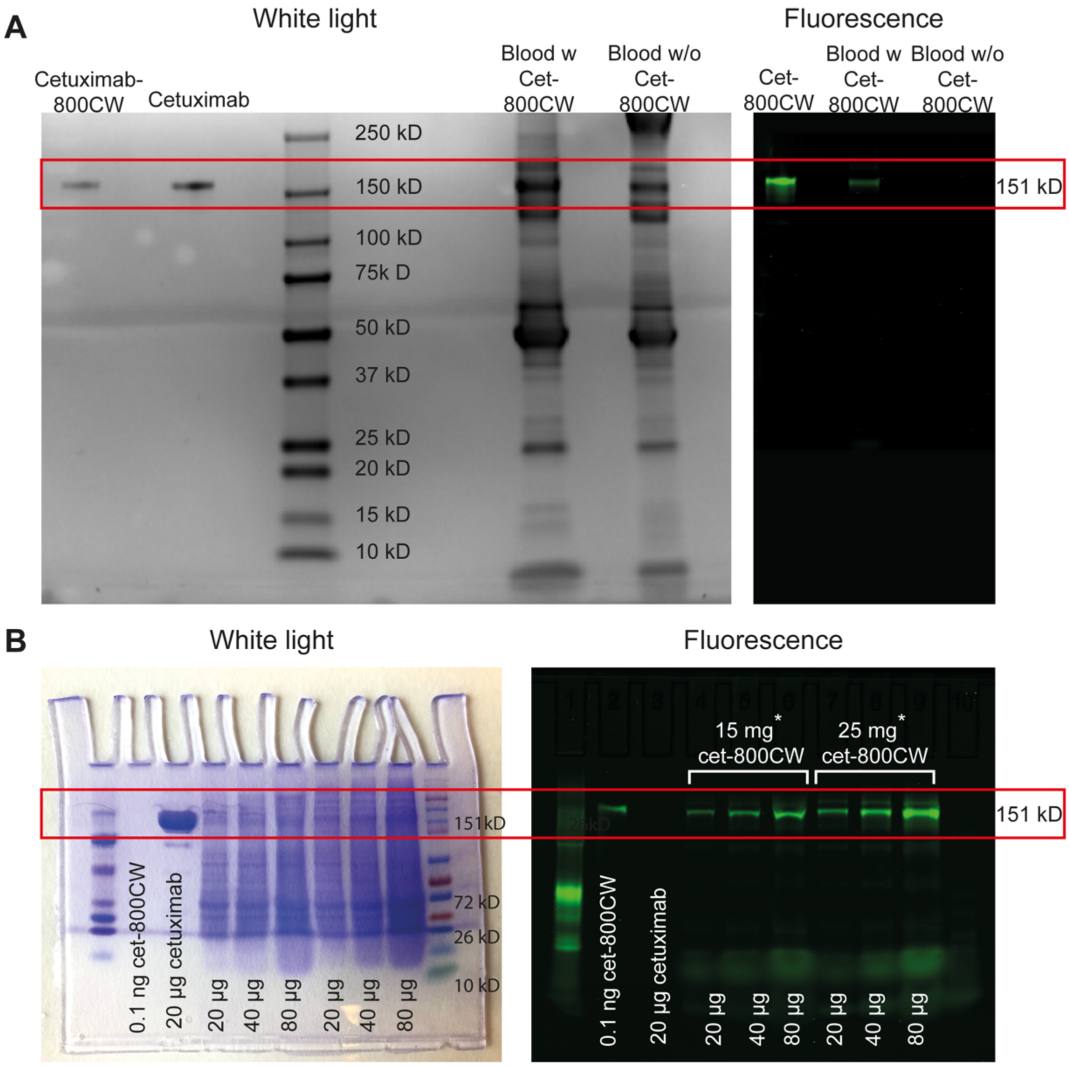

Figure S5 | Cetuximab-IRDye800CW integrity

Representative examples of cetuximab-800CW integrity (+- 151kD). A: SDS-PAGE of a blood sample collected four days after tracer administration. B: SDS-PAGE of a fresh-frozen tumor lysates collected immediately after tumor excision. * 75 mg cetuximab is administered one hour prior to cetuximab-800CW administration.

FIGURE S6

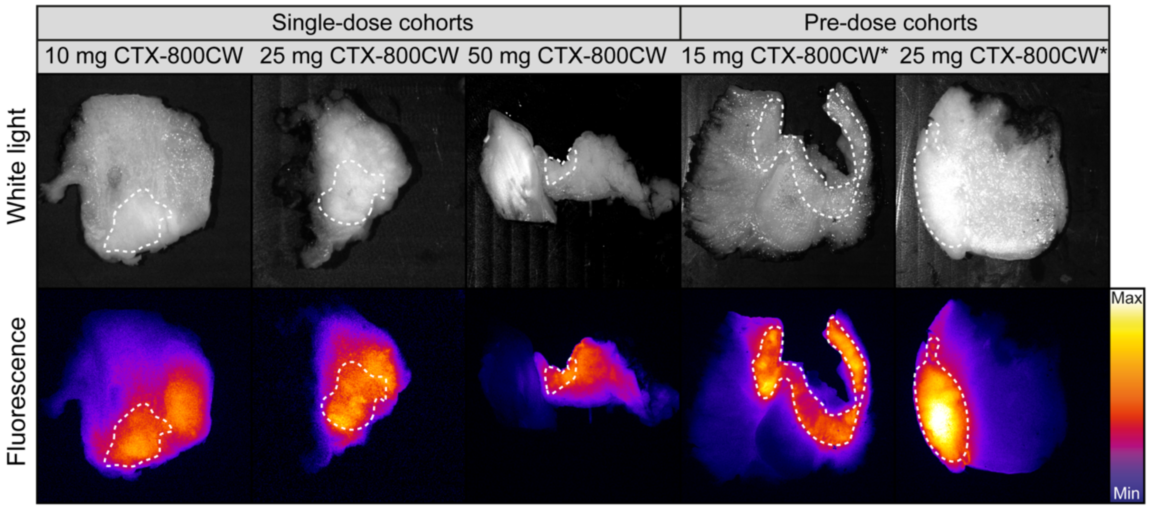

Figure S6 | Representative tissue slices containing tumor per dosing cohort

Representative images of tissue slices containing both tumor and non-tumor tissue per dose cohort. Histological proven tumor tissue is delineated with a dotted white line. Note the enhanced discrimination between tumor and non-tumor tissue and sharp delineation in the pre-dosed cohorts. Fluorescence intensities are scaled from minimum to maximum. *75 mg cetuximab is administered one hour prior to cetuximab-800CW administration. CTX: Cetuximab.

SUPPLEMENTAL VIDEOS

See separate file

Video S1 | Intraoperative visualization of a tongue carcinoma

Intraoperative visualization of cetuximab-800CW fluorescence of lateral tongue carcinoma. Patient is dosed four days prior to surgery with 75 mg cetuximab followed by 15 mg cetuximab-800CW.
